# Effects of the Modern Digital Information Environment on Maternal Health Care Professionals, the Role of Midwives, and the People in Their Care: Scoping Review

**DOI:** 10.2196/70108

**Published:** 2025-02-25

**Authors:** Elisabeth Wilhelm, Victoria Vivilaki, Jean Calleja-Agius, Elena Petelos, Maria Tzeli, Paraskevi Giaxi, Elena Triantiafyllou, Eleni Asimaki, Faye Alevizou, Tina D Purnat

**Affiliations:** 1 Department of Midwifery School of Healthcare and Care Sciences University of West Attica Athens Greece; 2 Department of Anatomy Faculty of Medicine & Surgery University of Malta Msida Malta; 3 CAPHRI Care and Public Health Research Institute Maastricht University Maastricht The Netherlands; 4 Faculty of Medicine University of Crete Rethymno Greece; 5 TH Chan School of Public Health Harvard University Boston, MA United States

**Keywords:** digital health, midwifery, misinformation, information-seeking midwifery, health information seeking, social media, medical misinformation

## Abstract

**Background:**

The digital information environment poses challenges for pregnant women and other people seeking care, as well as for their midwives and other health care professionals (HCPs). They can encounter questions, concerns, information gaps, and misinformation, which can influence health care decisions.

**Objective:**

This scoping review examines how HCPs are affected by the modern digital information environment including health misinformation, its effects on the women and people they care for, and its implications for care provision.

**Methods:**

English-language peer-reviewed literature, published from January 1, 2020, to May 31, 2024, with keywords related to midwifery, misinformation, and health equity collected and analyzed by a team of midwives and maternal care professionals and mapped onto a patient-centered conceptual model.

**Results:**

A total of 105 studies were ultimately included. Further, 95 papers identified specific digital information environment issues that affected clients; 58 specifically highlighted digital information environment issues impacting HCPs; 91 papers identified specific topics of common questions, concerns, misinformation, information voids, or narratives; 57 papers identified patient or population vulnerability; and 75 included mentions of solutions or recommendations for addressing a digital information environment issue around clients seeking care from midwives and other HCPs. When mapped onto the Journey to Health model, the most prominent barrier was access to care and information. Individual-level issues dominate the step related to knowledge, awareness, and belief, with more social norms and wider engagement appearing at steps related to intent. Client-specific themes dominate the left-hand side of the model and provider-specific issues dominate the right-hand side of the model.

**Conclusions:**

Misinformation, information voids, unaddressed questions and concerns, and lack of access to high-quality health information are worldwide prevalent barriers that affect both patients and HCPs. We identified individual, provider-level, health systems, and societal-level strategies that can be used to promote healthier digital information environments.

## Introduction

### Background

Pregnant women and parents-to-be are often exposed to a flood of information and advice not only from health care professionals (HCPs), but also from family, friends, and increasingly from various online sources [1]. However, not all of this online information is accurate and some might be deceptive [2], making it challenging for people to identify reliable sources of health information and to filter and act on reliable guidance regarding pregnancy, maternity, reproductive health, and other health topics [3]. The digital information environment—encompassing mobile phones, apps, social media, and websites—has profoundly influenced how people, including women and their partners in the perinatal period [4], seek and process health information. Inequities in access to health care and health information [5,6], influenced by social and commercial determinants of health [7], also affect how people’s health is affected by the digital information environment [8].

Midwives and other maternal HCPs need to better understand how the people they care for seek information online, including common questions, concerns, misinformation, and narratives that may be encountered. Although information about sexuality, reproductive health, and pregnancy may come from many sources, and people may seek offline information and care from other professionals and are influenced by social and support networks [9], the digital environment is now a leading source of information [10,11]. This understanding is critical, as it can impact the HCP-client relationship [12] and is especially critical for midwives.

Midwives strive to provide woman-centered care, with respect for autonomy, choice, and control. The midwife-woman relationship has trust as its foundational basis. Evidence-based midwifery provides for deliberative exchanges for shared decision-making to establish the wishes, needs, and preferences of women, and to develop care plans that can address the needs of women, ultimately empowering them, ensuring quality care for women and newborns, and safeguarding their health and well-being [13]. The digital information environment can influence all aspects of the unique relationship between midwife and woman and can influence downstream effects of health-seeking behaviors and decision-making and how women navigate maternity care systems; in addition, it can positively or adversely affect health outcomes [14].

It is particularly important to consider that the health care relationship in the perinatal period covers the years before, during, and after pregnancy, and often multiple sequential pregnancies can result in many years of almost continuous interaction. This is a period of change as the person becomes pregnant and a parent, and develops new identities and behaviors that last a lifetime and affect all health decisions for children, mothers, and entire families [15]. Midwifery-led care, according to the International Council of Midwives [16], includes the core competency to “adapt to and adopt new and emerging technologies that have been proven to enhance midwifery practice and care.” Understanding the modern digital information environment is essential to fulfilling this competency, and as the world becomes more digitally connected, its impact on people seeking care and their midwives and other maternal HCPs will only grow.

In this review, we catalog and describe how midwives and other maternal HCPs are affected by the modern digital information environment, including health misinformation, its effects on their clients, and the implications it has on care provision. The digital information environment affects both HCPs and their patients [17,18], but has differing effects on each and influences the care providers give, which is why understanding these interactions holistically is essential for improving health care. Therefore, we will use a model that encompasses the patient experience and the social and digital information landscape, as well as health care access in the review. The review also includes research that is focused on maternal HCPs beyond midwives to reflect the maturing nature of research in this field.

Based on previous literature on social media [19], digital ecosystems in health care, and the information environment in the context of health [20], we define the digital information environment as the online spaces where information—including news, social media content, and other digital material—is produced, curated, shared, discussed, and amplified across digital platforms and devices. However, access to both information and health care is not equitable for all. This review also focuses on vulnerable populations, or people who experience inequities in accessing health care, including migrants, people living in poverty, individuals with disabilities, and those with low literacy, to better understand how these groups are affected by health misinformation and the challenges within the digital information environment. Given that different HCPs have different terminology for people under their care—such as clients, patients, or parents-to-be—and this review includes papers from many medical and health disciplines, terms may be used interchangeably.

### Why Focus on Midwives and Their Relationship to Their Clients?

This review will map available evidence and help inform the development of strategies for midwives to help their clients better cope with the modern digital information environment, promote inclusive communication, build trust, and more successfully encourage clients to follow health care advice, therefore improving client health outcomes. Developing a more trusting and effective midwife-client relationship can also help the clients maintain seeking health services for themselves and their children beyond the perinatal period.

The underpinning philosophy of the interaction between midwives and patients in the digital information landscape is based on trust, enabling effective collaboration in navigating modern health care challenges [21]. Both groups operate within the same digital ecosystem, but the ways they engage with and are impacted by information and disinformation differ significantly.

Women, often the primary consumers of digital health information, increasingly rely on tools such as websites, social media, and apps to seek knowledge about their health. While this access can empower them to take a more active role in their care, it also exposes them to misinformation, conflicting advice, and volumes of iatrogenic content. The result can be confusion, anxiety, and even decisions based on unreliable sources [22].

Midwives, on the other hand, engage with the same ecosystem as intermediaries and guides. They must stay informed about the evolving digital health landscape to effectively filter, interpret, and contextualize the information women encounter [23]. Their role is to identify credible resources, dispel misinformation, and integrate digital health content into evidence-based midwifery care. This dual role of interpreter and gatekeeper reinforces trust between midwives and women, as midwives ensure that the information women rely on is accurate and applicable to their individual needs.

Moreover, the way digital information impacts midwives and women is shaped by their priorities and capabilities. For women, digital tools often serve as an entry point to health education, providing a sense of autonomy but also raising questions and concerns [24]. For midwives, these tools are professional tools [25], used to enhance shared decision-making and to address women’s concerns in a way that fosters trust and collaboration. While women may experience information as fragmented or overwhelming, midwives transform the digital landscape into a resource for empowerment.

Disinformation poses a unique challenge in this ecosystem. Women may encounter intentionally misleading content that exacerbates mistrust or undermines their confidence in professional advice [26]. For midwives, disinformation adds complexity to their role, requiring them to not only correct falsehoods but also rebuild trust when it has been eroded by inaccurate digital narratives. This dynamic underscores the critical importance of midwives as trusted navigators in the health care journey.

Trust extends beyond individual interactions to midwives’ broader advocacy for equitable access to credible digital resources. Systemic challenges, such as the digital divide and disparities in digital literacy, disproportionately affect women, especially those from underserved populations such as migrant mothers. Midwives address these inequities by guiding women toward accessible, understandable, and reliable health information. This advocacy, combined with evidence-based midwifery practices and critical thinking skills fostered through midwifery education, further equips midwives to support women effectively in the digital era [27].

Ultimately, the differentiated dynamics between midwives and women in the digital information ecosystem highlight the complementary roles they play. By synthesizing digital information, midwifery expertise, and trust, midwives ensure that women feel supported, informed, and confident as they navigate their health care journey [28].

### Review Questions

This review aims to identify the facilitators and barriers that the modern digital information environment presents to midwives and the care they provide to their clients.

The key questions to answer in this review are: (1) How has the modern digital information environment affected midwives? (2) What common types of health misinformation are midwives asked to address? (3) How has the digital information environment affected clients’ information and health-seeking behaviors?

## Methods

### Framework for Search and Analysis

This review adapted the UNICEF (United Nations International Children’s Emergency Fund) Journey to Health model to map factors specific to HCPs and clients that surface in the review’s included papers [20]. This framework is used to conceptualize the health care–seeking journey of individuals and the care provision journey of HCPs. These factors are linked to the digital information environment, including health misinformation, which may influence health information-seeking behaviors, care-seeking, and health care provision.

**Figure 1:**
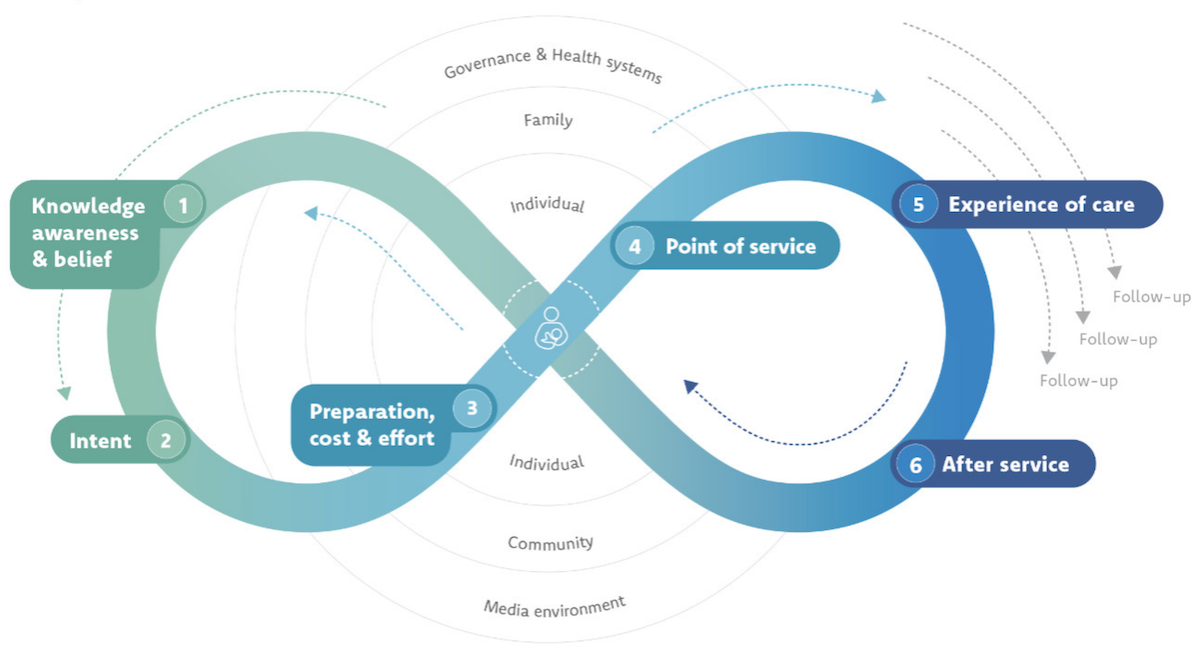
UNICEF’s Journey to Health model, which illustrates the 6 steps a patient or client takes in seeking health care and the multilevel factors that influence this journey. This review maps data extracted from included papers onto this model. The Journey to Health is licensed under the Creative Commons Attribution-NonCommercial-ShareAlike 3.0 IGO (CC BY-NC-SA 3.0 IGO) license. UNICEF: United Nations International Children’s Emergency Fund.

As shown in Figure 1, the model imagines the journey a patient or client undertakes to seek health care [29], and conversely, the HCP’s own journey in providing care, with individual-level and informational attributes on the left side of the diagram and more health service and care provision aspects found on the right side. For a patient or client to fully complete a course of recommended care, such as antenatal visits, they need to complete this journey many times but may encounter barriers that can make this journey harder, or enablers that make it easier. HCPs are also situated along this journey on the right-hand side at the point of service and heavily influence the experience of care and after-service for clients. This model also allows for the digital information environment effects on the HCP and the individual to be captured in the context of larger systems that shape their interactions. Any intervention or strategy—individual, community, health system, societal, or systemic—identified to manage the digital information environment would be included and mapped on the model.

The domains of inquiry during the analysis process, aligned with this framework, include themes of digital information environment barriers and enablers to clients seeking health care, midwives, and other HCPs in providing care, and what strategies or interventions were mentioned to overcome the identified digital information environment issue.

### Search Strategy

The domain being studied is digital information environment components, particularly health information, questions, concerns, information voids, narratives, health misinformation, and disinformation in online spaces [30], which can affect a person’s perceptions and health care decisions. In this review, the focus was on midwives but aimed to broaden the scope also to those HCPs who provided maternal health care, people in the perinatal period, and equity considerations.

Peer-reviewed papers were identified through databases including PubMed, Scopus, Web of Science, Science Direct, Cochrane Library, Embase, and Google Scholar (specifically review papers).

The search strategy was developed by identifying keywords from relevant recent publications and identifying additional terms associated with midwives and misinformation, social media, information environment, digital health, health information, health equity, and other related Medical Subject Headings (MeSH) keywords. Search using such an approach yielded very few papers, likely due to inconsistent coding of concepts related to misinformation, information environment, and interpersonal communication challenges in a health care setting. Therefore, the keyword string was reduced to broaden the search and cast a wider net for potentially applicable papers from the information environment point of view. At the same time, terms related to midwives and pregnancy were introduced to focus the search results on midwives and their clients and reduce the number of results that were not relevant to this setting and were being returned when we used broader terms referring to HCPs. This exercise resulted in the search string provided in the following paragraph and uncovered a more diverse array of relevant papers from health care journals, social science, social media, the internet, and technology.

The final search string was modified to fit the parameters of each scientific literature database: ((pregnancy) AND (midwife) AND (misinformation) AND (communication OR question* OR concern*)) AND equity.

To be included, papers were required to be published in peer-reviewed journals in English between January 1, 2020, to May 31, 2024, in addition to other criteria (Table 1). This timeframe was chosen due to the rapidly changing digital information environment, especially during and shortly following the global COVID-19 pandemic, which saw major social media platforms weaken content moderation against health misinformation [31], TikTok grow as a leading source of health information for women [32], health care provision shift to include telehealth and increase in health information-seeking online [33,34], and misinformation identified as a growing public health concern worldwide [35,36].

**Table 1:** Summary of inclusion and exclusion criteria for papers in this scoping review focused on midwives, the people they care for, and the modern information environment.

	Included	Excluded
Article type	Original research, reviews, meta-analyses, or case reports published in peer-reviewed research journals	Opinions and commentaries, gray literature, preprints, duplicates, or book chapters
Methodology	Can include any methodology or type of research (qualitative and quantitative)	N/A^a^
Geography	Any country or region	N/A
Language	English	Not in English
Publication date range	January 1, 2020, to May 31, 2024	Outside of January 1, 2020, to May 31, 2024
Full text available?	Yes	No
Related to digital information environment? (eg, includes social media, apps, and internet)	Yes	No
Relevant to maternal health professionals? (eg, includes midwives or OB-GYNs^b^ as health providers or pregnant women as the focus population)	Yes	No
Related to health equity? (eg, discusses disparities in information or health care access and use for different populations)	Yes	No

^a^N/A: not applicable.

^b^OB-GYN: obstetrics and gynecology.

### Analysis Team Setup

The screening and analysis process was undertaken by a multidisciplinary, multinational team of health care and public health–focused academics and professionals, including several midwives. This ensured that the review of the literature to inform midwifery-led care was also led by midwives and that the extracted data and conclusions were correctly interpreted from a health care lens. A standard operating procedure (SOP) was developed and the team was trained on processes and tools to follow SOPs. The team collaborated at every stage, including screening, data extraction, analysis, and synthesis, conducting weekly check-in meetings discussing results, reviewing all entries and reconciling disparate judgments on particular papers, spot-checking one another’s work, and updating SOPs and definitions to ensure consistency of screening and analysis processes.

### Screening

Searches for papers for inclusion in the review were conducted in all identified peer-reviewed literature databases in English using defined date ranges and MeSH search terms. These papers (n=895) were then exported into Paperpile where some records were removed before screening, including duplicate records (n=29), non-English ones, those outside of the desired date range, or those initially flagged as not from a peer-reviewed journal (n=297). The remaining records (n=523) were screened, of which, 25 reports were not retrieved, and 498 were assessed for eligibility. Based on a full review of each paper, reports were then excluded for not being peer-reviewed (n=78), not being published within the defined date range (n=17), not being relevant to midwives (n=99), and not including an aspect of digital information environment (n=350). Ultimately, 105 papers were included in the review.

### Data Extraction and Thematic Analysis

After the final list of studies to be included for review was compiled, they were loaded into a shared spreadsheet. The analysis team members then received randomized selections of the papers for further data extraction, coding, and thematic analysis. Each assigned paper was thoroughly read, reviewed, and discussed by the team. Any disagreements in judgments were resolved collaboratively before proceeding to the next stage. Papers were assessed for quality and qualitative papers were scored using a modified RATS (relevance, appropriateness, transparency, soundness) scoring framework.

In the data extraction phase, a PICO (population, intervention, comparison, and outcome) framework was used to extract and record the following information for each paper: (1) primary population of focus (HCPs), (2) stage of reproductive journey, (3) status and identities of population of focus that impact health equity, (4) country, (5) type of study, (6) link with COVID-19, (7) aspect of digital information environment investigated, (8) (if included) interventions or strategies to address digital information issue, and (9) intervention effects.

For the field (7) aspect of digital information environment investigated, the codes were developed based on a preparatory literature search and the papers analyzed: information-seeking, coping or support-seeking, community-seeking, information overload, information or news exposure, misinformation (inaccurate information on products, service, health information, or health guidance), disinformation (deliberately created inaccurate information on products, service, health information, or health guidance spread for political or financial gain), expressing questions or concerns, digital-mediated social issues (eg, bullying, harassment, stigma, or doxxing), social media use, app use, social media content (eg, memes, hashtags, videos, or images), deceptive marketing or advertising, communication with HCP, infodemic, and other.

### Coding Process Across Domains

In the coding stage, the papers were coded for specific domains, with key information from relevant sections of papers extracted and added to the spreadsheet. These domains included: how the identified digital information environment issue affected clients, how it affects HCPs, and topics of common questions, concerns, misinformation, information voids, or narratives identified. Considerations related to patient or population vulnerability and any recommendations or recommended solutions to address the infodemic environment issue were also captured and coded.

### Synthesis of Results

In this phase, analysts reviewed all 105 papers according to the domains listed above. To facilitate thematic grouping and visualization, the data were transferred to Miro, a digital whiteboard, where sticky notes were created, shared, discussed, and organized into themes during videoconference calls. Identifying new themes came from grouping similar ideas together [37] and identifying visually what ideas were on their own (or outliers) and placing similar groupings of ideas close to one another or between 2 different ideas that are linked. For example, COVID-19 as a theme is often related to the vaccines theme and fertility theme. Then, after the group discussion and organization of ideas on sticky notes, a template was used to help consolidate observations for each domain. Themes were then mapped on the Journey to Health model to provide a visual perspective on where along the journey the theme fits best and discussed with the analysis team to inform implications for HCP practice.

## Results

### Papers by the Numbers

**Figure 2:**
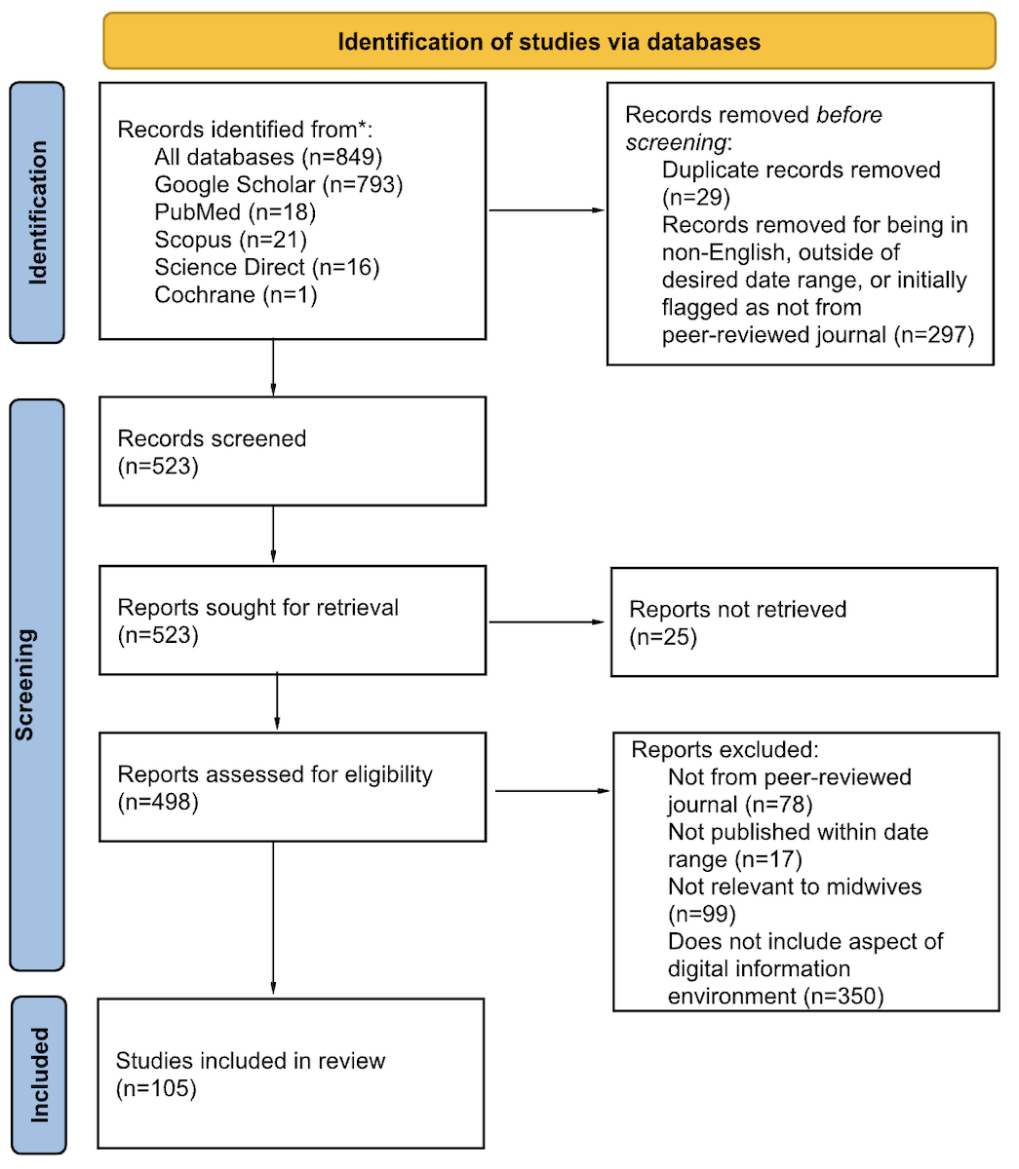
Identification of studies via databases included in this review focusing on midwives, their clients, and health misinformation and related digital information environment issues, adapted from the PRISMA 2020 flow diagram. PRISMA: Preferred Reporting Items for Systematic Reviews and Meta-Analyses.

The PRISMA-ScR (Preferred Reporting Items for Systematic Reviews and Meta-Analyses extension for Scoping Reviews) checklist is available in Multimedia Appendix 1. Of 105 papers (Figure 2) included in the review, 28 papers were a type of review, systematic reviews, or meta-analyses, 91 papers discussed a vulnerability or aspect of health equity, 74 papers included multiple countries, 37 were COVID-19–focused, 46 included misinformation as the major digital information environment issue, 48 had suggested interventions or solutions with no papers including intervention effects, 75 had related recommendations or solutions, and 91 included topics of common questions, concerns or misinformation identified. Only 3 papers were solely focused on midwives versus broader categories of maternal HCPs. Additional information about included papers is summarized in Table 2.

**Table 2:** Selected characteristics of included papers in this scoping review focused on midwives, their clients, and digital information environment challenges.

Specific characteristic	Number of papers (of 105 papers included in review), n (%)
Paper a review (eg, review, systematic review, or meta-analysis)	28 (26)
Paper related to a health vulnerability or health equity issue	91 (86)
Papers included multiple countries	74 (70)
Papers that were COVID-19–focused	37 (35)
Papers that included misinformation as a major digital information environment issue	46 (43)
Papers that included specific interventions or strategies to address digital information environment issue	48 (45)
Papers that included intervention effects	0 (0)
Papers that included recommendations or solutions to address digital information environment issue	75 (71)
Papers that included topics of common questions, concerns, or misinformation identified	91 (85)
Papers that were specific to midwives compared to other HCP^a^ types or non-HCP audience	3 (2)

^a^HCP: health care professional.

### Results by Domain

#### How Identified Digital Information Environment Issues Affected Clients

Of 105 papers, 95 papers identified specific digital information environment issues that affected clients. The most common topics were COVID-19, vaccine hesitancy, access, decision-making and care-seeking, breastfeeding, experience of care or HCP relationship, poor quality information, social norms, lack of trust, information overload, anxiety, peers, gender, and self-esteem or body image.

Several common and cross-cutting themes emerged for this domain. These included mental and cognitive barriers where clients expressed anxiety, experienced information overload, and had difficulty determining information quality and distinguishing between accurate and inaccurate information. Information voids also featured, where clients could not find the information that they were looking for from online resources or found the messaging confusing or mixed. This sometimes led to reliance on lower quality available information or misinformation. Informational barriers, such as lack of access to health information, including internet access and information available in appropriate formats, language, and cultural relevance could make people more prone to misinformation. Negative effects on behavior were also documented, where misinformation exposure can lead to attitudes and behaviors that put clients’ health at risk. Finally, trust in HCPs was a barrier where poor health care encounters and stigmatizing experiences aggravated clients’ mistrust in HCPs who then sought information and care elsewhere, especially from online spaces where they felt more socially connected and supported.

**Figure 3:**
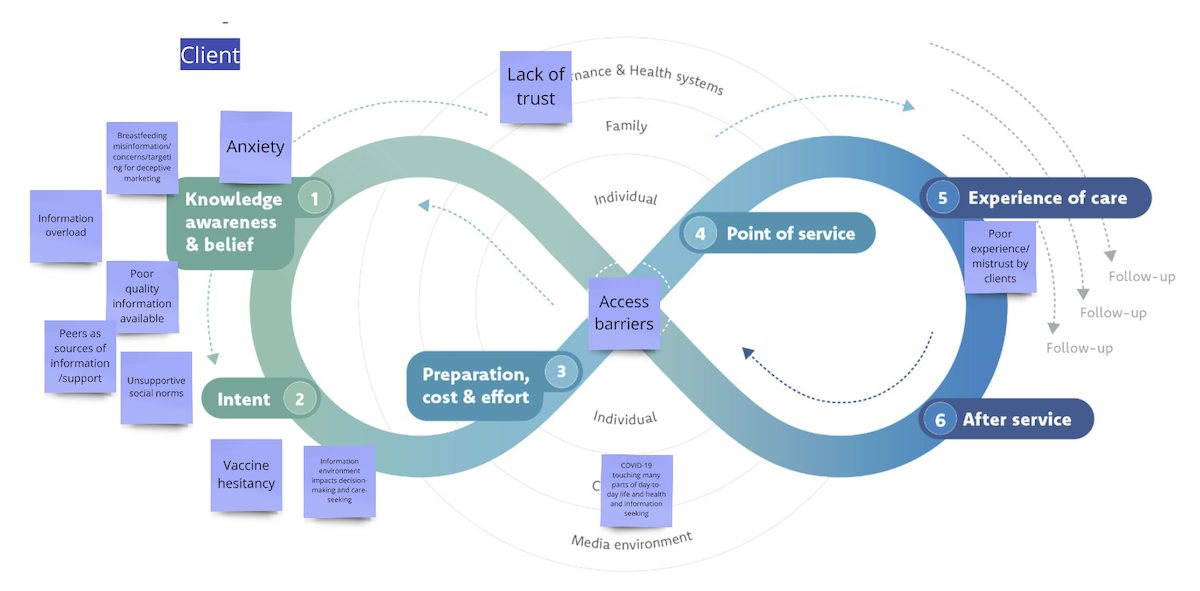
Sample screenshot of Journey to Health with major themes related to client barriers and enablers related to digital information environment challenges mapped, clustering around the (1) knowledge, awareness, and belief and (2) intent stages, which can influence care seeking in later steps of the journey.

As shown in Figure 3, on the Journey to Health model, the clustering of themes in earlier stages of the journey highlights individual-level factors, information environment issues affecting knowledge and decision-making about health, and therefore making the rest of the journey and advancing to (3) preparation, cost, and effort and beyond more difficult. Digital information environment challenges can place informational barriers in the way of health care seeking. Access barriers further hindered the client journey, suggesting that some people who need care are not able to obtain it, and for those who do—for (5) experience of care—poor-quality interactions with HCPs by clients make it less likely they will trust them further or return.

At a higher level of the socioecological diagram encompassing the journey, trust, or lack thereof, played a prominent role in influencing client attitudes toward health providers, their advice, and larger health care systems. COVID-19 also played a disruptive role, affecting the information environment and how care was offered, resulting in a chaotic information environment often seeding confusing information, providing mixed messaging, and making it difficult to communicate with HCPs.

#### How the Identified Digital Information Environment Issue Affected HCPs

Of the 105 papers reviewed, 58 specifically highlighted digital information environment issues impacting HCPs.

The most common topics identified included poor provision of care, stigma, the impacts of COVID-19, HCPs as both a source of misinformation and as a means of addressing it, lack of accurate guidance for HCPs, communication challenges, alternative information-seeking behaviors where clients circumvented their HCP, trust, the role of digital apps, and direct negative impacts on HCPs.

Across papers mentioning HCP-side challenges, several themes emerged. HCPs had dual-sided roles in information provision, some actively promoting misinformation and others addressing misinformation on a variety of topics, especially during the COVID-19 pandemic. COVID-19 also was the catalyst for the rapidly changing digital information environment, significantly accelerating challenges within the digital information environment for HCPs, driving greater digitization, the growth of telehealth and mobile health (mHealth), and changing client expectations around addressing misinformation. HCPs reported that misinformation was not only a challenge to providing health care but also a factor that affected their clients’ attitudes and decision-making. HCPs also reported a lack of training or professional guidance on how to address misinformation and other challenges within the digital information environment, including improving their own digital literacy and evaluating mHealth apps used by clients. Misinformation contributed to less emphatic care where HCPs stigmatized or discriminated against clients based on misinformation. When clients lack access to or mistrust HCPs, they will seek and act on health information elsewhere, which can make it difficult for HPCs to understand what health information (or misinformation) influenced their clients and their resulting health decisions.

Themes were mapped onto the Journey to Health model, spanning most stages from (1) knowledge, awareness, and belief to (2) intent; (3) preparation, cost, and effort; (4) point of service; and (5) experience of care. Several themes clustered about the point of service and experience of care, indicating that digital information environment challenges such as misinformation affected health care provision. Larger social and systems forces related to the information environment and mistrust in HCPs and health systems by patients and COVID-19 as a major event disrupting health care also played a role in changing how care was provided.

#### Topics of Common Questions, Concerns, Misinformation, Information Voids, or Narratives Identified

Of 105 papers, 91 papers identified specific topics of common questions, concerns, misinformation, information voids, or narratives identified.

Common topics included COVID-19, vaccines, reproductive health, pregnancy, breastfeeding, family planning, LGBTQ+ (lesbian, gay, bisexual, transgender, queer, and other identities), baby, labor and birth, nutrition, obesity, and stereotypes about groups of people and mental health. These topics were not just misinformation but also reflected topics where there was confusion, lack of information, misperceptions, or questions and concerns. These are topics that patients expected their providers to be able to address but reported feeling unsatisfied with given HCP-provided information and advice.

There were only two themes that emerged beyond specific topics, including issues related to information seeking between (1) knowledge, awareness, and belief and (2) intent and lack of access before (4) point of service on the Journey to Health. Both are about seeking information and health care, while the topical items were not specific to any stage of the journey.

#### Considerations Related to Patient or Population Vulnerability

Of 105 papers, 57 papers identified patient or population vulnerability.

The types of vulnerability discussed included multiple vulnerabilities, migration or refugee status, religious or ethnic minority status, adolescence, mental health vulnerabilities, disability, poverty, LGBTQ+ status, and adverse life experiences, among others. COVID-19 also highlighted the vulnerabilities of specific groups, including those with pre-existing health conditions and pregnant people.

The most common topics were access, information seeking, trust, social marginalization and stigma, literacy, and risk perception.

Emerging themes included access barriers, which included legal or policy, social, cultural, and physical access challenges to health information and care. Access to care is made more difficult if someone has a vulnerability or comes from a vulnerable population. Information seeking, shaped by literacy, identity, and access also featured as a theme. People will seek health information from sources they trust, which often includes family, friends, and larger peer groups where they share identity and community.

Themes mapped onto the Journey to Health around vulnerabilities clustered around (1) knowledge, awareness, and belief and (2) intent. Vulnerabilities can affect individual-level and social processes and barriers in earlier stages of the journey can limit access to care.

#### Recommendations or Solutions Are Presented to Address Digital Information Environment Issues

Of 105 papers, 75 included mentions of solutions or recommendations for addressing a digital information environment issue around clients seeking care from midwives and other HCPs. These focused on system-side interventions or solutions compared to more general societal or government-level interventions, such as technology platform regulation, content moderation, or fact-checking, which are well documented in the literature elsewhere [38,39].

Cross-cutting themes focused on addressing skills and performance gaps by HCPs. This included improving HCP communication with strategies such as health education and counseling. Solutions were often designed to improve the quality of communication, language clarity, and inclusiveness, especially for populations that have experienced stigma or discrimination, such as people with disabilities, migrants or refugees, and LGBTQ+ people. Improving knowledge and communication on related topics, such as nutrition, can also address patient concerns. Training HCPs was also a common theme, with suggested topics on how to address misinformation, communicate more clearly, and receive training to better address vaccine hesitancy. HCPs should also be trained to give more inclusive and emphatic care to LGTBQ+ people. HCPs can also address health misinformation, using techniques such as prebunking, improving resilience and health literacy in clients, and training HCPs to address misinformation successfully.

At the systems level, more work can be done to improve risk communication by improving communication by health authorities and within families, During emergencies, it is important to evolve messaging as more information becomes known, especially for pregnant women and people in the perinatal period. Several papers suggested clarifying health systems policies such as by formalizing how HCPs are trained or expected to address misinformation, encouraging more research on health information, digital environment, and patient decision-making, and ensuring that health professionals adhere to ethics regarding product marketing and sponsorship. Improving access to health care through improved access to information could be accomplished by increasing the availability of information and enhancing access to information tools and technologies (eg, mHealth apps), particularly for patients who have historically faced inequitable health care access. Strengthening digital engagement was also recommended by promoting HCPs to use digital spaces to educate and connect with clients as trusted voices and improve the quality of available health-related social media content.

Additional themes focused on community-level strategies. Increasing community outreach, participation, and cocreation could help address the lack of tailored information or services and help enhance patient communication and care. This can be carried out by improving health system–community linkages and strengthening communications beyond pregnant women, including their communities and networks as well. Mainstreaming culturally competent communication and care by HCPs could be accomplished by policies and training to improve HCP cultural competency to better care for diverse populations. This could provide more culturally appropriate health information and support midwives and HCPs in reducing stigma by providing trauma-informed care. Finally, at the individual level, health and digital literacy could be improved through health education programs for the public and via HCP in appointments with clients.

When mapped onto the Journey to Health model, recommendations and solutions predominantly cluster on the left side, associated with individual-level knowledge, awareness, and intent. However, the solutions and recommendations cut across individual-level, provider-level, societal, health systems, and government or policy levels in recognition that the complex information environment’s effects may need to be addressed through multiple levels.

#### Putting It All Together: Mapping Across All Domains and Themes

**Figure 4:**
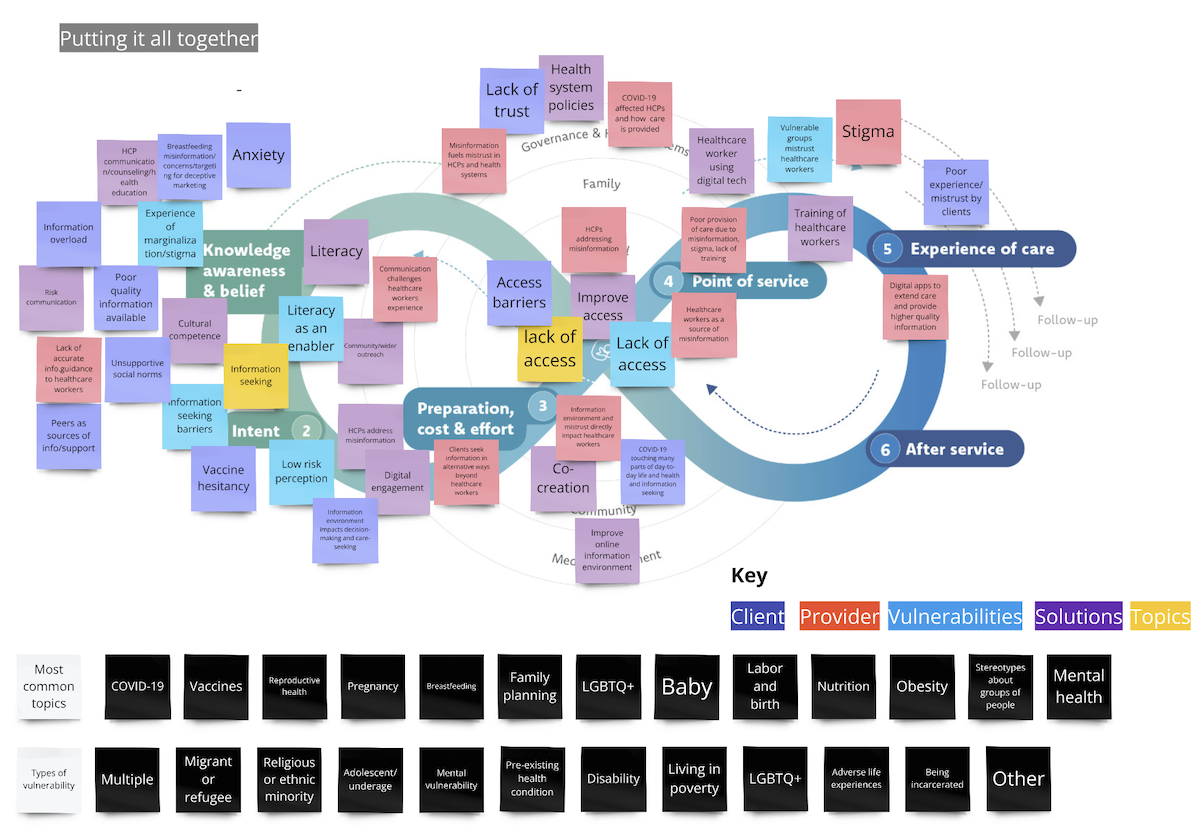
Screenshot of Journey to Health with major themes, common topics, and vulnerabilities mapped to address digital information environment issues.

After each domain and major theme were mapped on the Journey to Health, all themes, still color-coded, were mapped onto the model (Figure 4). Themes were heavily clustered in steps 1-5, with access (or lack thereof) appearing consistently for all themes except for the provider. Individual-level issues dominate at step (1) knowledge, awareness, and belief, with more social norms and wider engagement appearing at step (2) intent. Client-specific themes dominate the left-hand side and provider-specific issues dominate the right-hand side.

Screenshots of the Miro whiteboard with details of the analysis of all domains are available in Multimedia Appendix 2.

## Discussion

### Principal Findings

The COVID-19 pandemic, which occurred during the period of data collection for this review, featured heavily in the included papers, where 35% (37/105) were directly related to the virus and its impacts on health systems and societies. Concepts such as “infodemic” [40] emerged and gained prominence, as did the role of misinformation in affecting health care provision and health care seeking worldwide [41]. Telehealth and digital communication to provide care became more common, and in the context of mobility restrictions, people sought more information via their mobile devices. As a result, the digital divide and inequitable access to health care for some vulnerable groups became prominent. These inequities predated COVID-19 but the pandemic highlighted new concerning dimensions where inequitable access to credible, accurate health information in the digital information environment adversely impacted health.

The review illustrates that the digital information environment exacerbates existing inequalities in access for clients and patients. HCPs struggle to overcome these challenges, particularly due to a lack of training or supportive workplace policies. Access to health care is often the first barrier faced by vulnerable populations, but even when access is attained, it does not guarantee that empathetic, culturally competent care will be provided. HCPs can struggle to adequately address patient questions, concerns, or misinformation effectively.

Individuals experiencing vulnerabilities have to deal with the dual challenges of limited access to care and credible health information. This can make them more vulnerable to exposure to low-quality information or misinformation that could negatively influence their perceptions or health behaviors. The COVID-19 pandemic provided many examples, including vaccine hesitancy among pregnant people based on mixed messaging and misinformation [42], some communities such as migrants, refugees, and other minority ethnic groups not seeking health advice reflecting their information needs, language, or values [43]. HCPs did not always meet these information needs, nor did wider health systems [44].

Some HCPs are actively addressing misinformation while others spread it, reflecting a lack of consistency in standards, training, and expectations of HCPs by health systems. However, HCPs report that since the COVID-19 pandemic, misinformation is affecting how they provide care, and that it is no longer just an inconvenient side effect of living in the modern age.

Health misinformation is widespread on a large variety of topics of interest to people in the perinatal period [45]. How people sought it was based on who they trusted and what information sources they had access to, including HCPs. In communities that did not trust HCPs or had limited access to them, people sought out information from trusted networks including family, friends, and online support groups to help them cope, and find community and information that they could not easily obtain elsewhere. For some vulnerable groups, such as LGBTQ+ people, health information was sometimes entirely missing. This information void also affects HCPs, who may provide misinformation or provide poor, nonaffirming care to their clients.

HCPs need to broaden their knowledge of the related and trending topics their clients may have questions and concerns about and not assume that clients are operating only on credible information sources on which to base their health decisions. HCPs also should recognize that they compete for their patients’ attention and trust against more emotionally compelling, slickly packaged, and immediately accessible social media content.

Health literacy emerged as a meta-theme based on numerous themes about information quality, culture, accessibility, and format—ideas that surfaced for every domain. Specifically, components of literacy [46] appeared on the individual level for clients about context, skills, knowledge, and psychological factors. Importantly, lack of digital literacy skills was not simply a challenge identified among patients, but also among HCPs, where lack of formal training or unclear workplace norms exacerbate this skills gap.

However, effective communication with patients takes more than a connection and literacy skills. Dozens of papers highlight issues of racism, stigma, discrimination, poor care, or care that even aggravates pre-existing trauma that HCPs can project onto their patients. Patients who already experience multiple vulnerabilities also face much higher hurdles to arrive at an HCP’s office, if misinformation and access issues have not dissuaded them from trying to seek access. Treating them with care and respectful communication is a critical factor for them to follow health care advice and seek care in the future.

Intercultural competence and understanding the digital information environment and how it affects their patients are no longer optional for HCPs. Better interventions could be cocreated with patients, communities, and providers to address digital information environment challenges effectively. Such intervention could be better keyed to the information needs of vulnerable groups and take into account the complex web of information and social networks in digital spaces that are beyond the typical scope of HCPs. Given the widespread nature of health misinformation and other digital challenges, addressing them will require community-oriented solutions. Community-based solutions to combat misinformation could include cocreated interventions involving HCPs, patients, and community leaders, aimed at developing digital health literacy programs tailored to the specific needs of vulnerable groups, ensuring culturally relevant and accessible content.

### Strengths and Limitations

The strength of this scoping review is its systematic approach, providing a snapshot of current digital information environment challenges and how they relate to midwives, maternal HCPs, and clients, including during the COVID-19 pandemic. However, this review is not exhaustive and did not include languages other than English, additional scientific databases, or gray literature. In future reviews, the inclusion of non-English language studies and gray literature could enrich the analysis, offering a broader, more international perspective on how different countries and cultures are addressing digital misinformation in health care. Additionally, as MeSH terms related to misinformation are largely categorized under communication rather than medical or public health topics, there is a risk that some relevant papers may have been overlooked. In the initial development of search strings, the terms “midwife” and “pregnancy” were included to focus on the health professional type in focus and the health state in focus for this paper; however, the search terms also led to the inclusion of broader maternal health care provider categories, which led to the inclusion of papers that did not specifically target midwives but were potentially relevant to them. This synthesis acknowledges limitations in the assessment of quality and access to digital information. A scoping review approach was used to map out the current landscape, ensuring the inclusion of diverse perspectives while recognizing gaps in data. Future work should focus on addressing disparities in access to technology and digital literacy among different populations.

### Conclusion and Implications for Practice

The challenges presented by the digital information environment—misinformation, information voids, unaddressed questions and concerns, and lack of access to high-quality health information—are global issues. These barriers to optimal health are particularly detrimental for individuals seeking maternal health services, especially for those facing social, economic, or health vulnerabilities. Some barriers affect both patients and HCPs in achieving a healthier digital information environment that would promote accurate, accessible health information that people trust.

Despite these challenges, HCPs, especially midwives, are well-positioned to address misinformation and other information environment challenges. This is because HCPs are trusted sources of health information, including online. They also have pre-existing relationships with clients and communities and can extend relationships and continuity of care in digital spaces. HCPs can translate health guidance and evidence through more mediums, especially when digital “work” and tools are mainstreamed into care. HCPs can also empower clients with health education and promote digital health literacy. HCPs can also provide empathetic care with enhanced intercultural competencies through updated training.

However, to enable midwives and other HCPs to effectively address digital information environment challenges, more policy and health systems–level shifts are needed. HCPs should receive improved training in digital literacy, cultural competency, and caring for populations with specific vulnerabilities. Similar to how an HCP may ask questions related to identifying risk for intimate partner violence or about nutrition and dietary habits and tailor their advice and care accordingly, understanding how the digital information environment is affecting their patients may be a new type of risk assessment offered. All HCPs should also benefit from work environments that promote mental health, and provide needed training and additional informational support for serving diverse patient populations.

The digital information environment will continue to grow in influence and impact health care delivery and information-seeking behaviors. Therefore, HCPs should be better supported through training and inclusion in health visits to address misinformation and other related issues. Health systems must improve their communication strategies, particularly in digital spaces, to provide accurate, timely, and culturally relevant health information to diverse audiences. One promising approach is to encourage HCPs to play an active role in digital spaces, leveraging the trust patients place in them to educate the public, engage with specific communities, and address misinformation in online communities where official health communication might not reach. Furthermore, health systems must prioritize the development of digital literacy training for both patients and HCPs, and adopt policies that support culturally competent, empathetic, and inclusive care.

Although most papers included in this review focused on general groups of maternal HCPs rather than specifically midwives, there are key insights particularly relevant to midwives. Midwives have long-term relationships with their pregnant clients while also addressing the sexual and reproductive health needs of many groups of people, from adolescents to refugees to LGBTQ+ people. Midwives are well-positioned at the client level and community level to listen, educate, and engage in offline and offline spaces, and play a crucial role in addressing health-related questions, concerns, and misinformation. It is imperative to include digital literacy, social media, cultural competency, and interpersonal communication training more firmly into midwifery education and extending midwifery practices into digital spaces. This would help midwives promote the values of empowerment, respect, and collaboration in this new digital frontier and can help them better serve the needs of clients who are increasingly online and navigating complex digital health landscapes.

## Data Availability

The datasets generated or analyzed during this study are available from the corresponding author upon reasonable request.

## Appendix

Multimedia Appendix 1 PRISMA-ScR: Preferred Reporting Items for Systematic Reviews and Meta-Analyses Extension for Scoping Reviews.

Multimedia Appendix 2 Themes identified from analysis of information environment challenges to health care professionals and their clients; each Journey to Health graphic shows the mapping of themes for health care professional, client, topic, vulnerabilities, solutions, and a consolidated overview.

## Abbreviations

HCP: health care professional
LGBTQ+: lesbian, gay, bisexual, transgender, queer, and other identities
MeSH: Medical Subject Headings
mHealth: mobile health
PICO: population, intervention, comparison, and outcome
PRISMA-ScR: Preferred Reporting Items for Systematic Reviews and Meta-Analyses extension for Scoping Reviews
RATS: relevance, appropriateness, transparency, soundness
SOP: standard operating procedure
UNICEF: United Nations International Children’s Emergency Fund
