# Supplementary figures and images for: Effects of the Modern Digital Information Environment on Maternal Health Care Professionals, the Role of Midwives, and the People in Their Care: Scoping Review
Source: J Med Internet Res. 2025 Feb 25;27:e70108. doi: 10.2196/70108 (PMC11897670; doi:10.2196/70108)

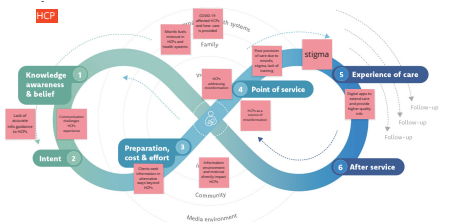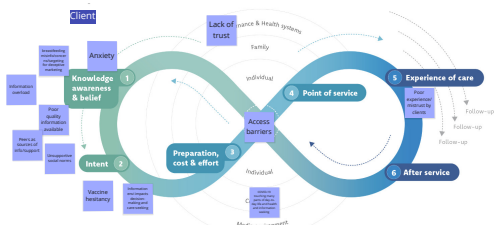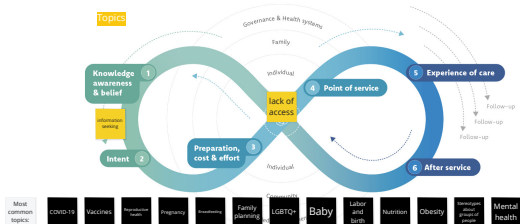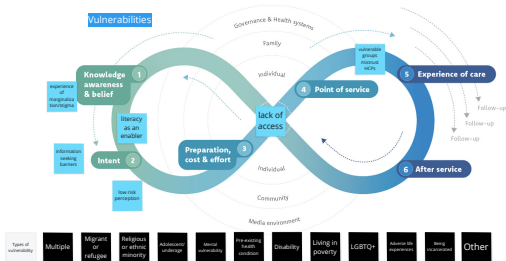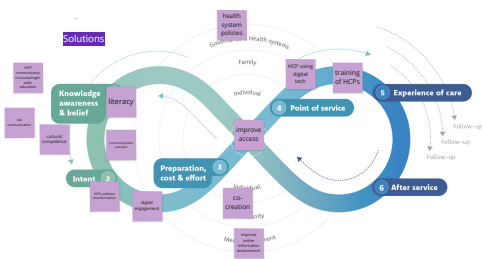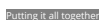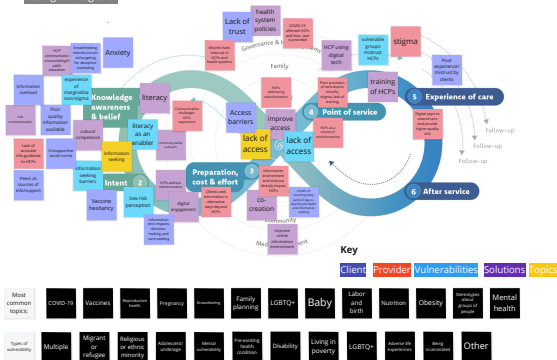

Supplement: Multimedia Appendix 2 [file jmir_v27i1e70108_app2.pdf]
